# Tumours of Oddi: Diagnosis and Surgical Treatment

**DOI:** 10.1155/1992/54935

**Published:** 1992

**Authors:** B. Nordlinger, B. Jeppsson, W. El-Khoury, L. Hannoun, P. Frileux, C. Huguet, M. Malafosse, R. Parc

**Affiliations:** 1 Centre de Chirurgie Digestive de l'Hopital Saint-Antoine 184 rue du Faubourg Saint-Antoine Paris F-75012 France; 2 Department of Surgery Lund University LUND S-221 85 Sweden

## Abstract

A retrospective review of 56 patients operated upon for tumours of Oddi was performed in order to determine optimal diagnostic and therapeutic procedures. Common presenting symptoms were jaundice (86%) and anemia (21%). Mean size of the tumour was 2.3 cm. Five tumours were benign and 51 were malignant. According to the classification of Martin, five were grade I: 10 grade II; 18 grade III; and 18 grade IV. Forty-seven patients underwent resection of the tumour: three local excisions for small benign tumors, six ampullectomies (followed in three by a Whipples’ procedure for recurrence) and 41 Whipples’ procedures. The hospital mortality was 5.3%, minor complications appeared in 21%. The overall five years survival was 41%. It was 75% in grade I, 50% in grade II, 40% in grade III and 10% in grade IV. The patients who received ampullectomies were alive with a follow-up of one, two and three years. All patients operated upon for a benign tumour were alive except one who died of cardiac failure. Ultrasonography and duodenoscopy are the most useful tests for the diagnosis of tumours of Oddi. Prognosis depends on the degree of infiltration of the duodenal wall and the presence of positive lymph nodes. Whipples’ procedure is best but ampullectomy can be used in elderly or poor risk patients. Malignant tumours of the ampullary region are infrequent and reported to constitute betwee 0.02 and five percent of all cancers of the digestive tract. With wider application of endoscopic techniques, there has been an increasing interest in this group of tumours during recent years. In the literature tumours of Oddi are usually reported in the group of periampullary tumours, including tumours of the ampulla itself, duodenal wall surrounding the ampulla, the distal part of the common bile duct and head of the pancreas. We have wanted to distinguish specifically the tumours of the ampulla of Vater and have adopted the term tumour of Oddi introduced by Marchal and Hureau.The sphincter of Oddi exactly delineates the junction between the bile duct, pancreatic duct and duodenum. We wanted to avoid using the anatomic term ampulla of Vater, since this structure rarely appears as an ampulla. This then excludes tumours in the head of pancreas, common bile duct above ths phincter of Oddi and tumours of the duodenal wall adjacent to the papilla. These tumours seem to behave differently from other pancreatic tumours, as they carry a different prognosis and need special attention. We have therefore reviewed retrospectively 56 patients with tumours of Oddi with special reference to diagnosis, histopathologic examination and surgical therapy.


*HPB Surgery*, 1992, Vol. 5, pp. 123–133
Reprints available directly from the publisher
Photocopying permitted by license only

© Harwood Academic Publishers GmbH
Printed in the United Kingdom

### TUMOURS OF ODDI: DIAGNOSIS AND SURGICAL TREATMENT

B. NORDLINGER, B. JEPPSSON*, W. EL-KHOURY, L. HANNOUN, P. FRILEUX, C. HUGUET, M. MALAFOSSE and R. PARC
*Centre de Chirurgie Digestive de l'Hopital Saint-Antoine, Paris, France*

*(Received 8 July 1991)*

A retrospective review of 56 patients operated upon for tumours of Oddi was performed in order to determine optimal diagnostic and therapeutic procedures.

Common presenting symptoms were jaundice (86%) and anemia (21%). Mean size of the tumour was 2.3 cm. Five tumours were benign and 51 were malignant. According to the classification of Martin, five were grade I: 10 grade II; 18 grade III; and 18 grade IV. Forty-seven patients underwent resection of the tumour: three local excisions for small benign tumors, six ampullectomies (followed in three by a Whipples' procedure for recurrence) and 41 Whipples' procedures. The hospital mortality was 5.3%, minor complications appeared in 21%.

The overall five years survival was 41%. It was 75% in grade I, 50% in grade II, 40% in grade III and 10% in grade IV. The patients who received ampullectomies were alive with a follow-up of one, two and three years. All patients operated upon for a benign tumour were alive except one who died of cardiac failure. Ultrasonography and duodenoscopy are the most useful tests for the diagnosis of tumours of Oddi. Prognosis depends on the degree of infiltration of the duodenal wall and the presence of positive lymph nodes. Whipples' procedure is best but ampullectomy can be used in elderly or poor risk patients.

Malignant tumours of the ampullary region are infrequent and reported to constitute betwee 0.02 and five percent of all cancers of the digestive tract[1]. With wider application of endoscopic techniques, there has been an increasing interest in this group of tumours during recent years. In the literature tumours of Oddi are usually reported in the group of periampullary tumours, including tumours of the ampulla itself, duodenal wall surrounding the ampulla, the distal part of the common bile duct and head of the pancreas. We have wanted to distinguish specifically the tumours of the ampulla of Vater and have adopted the term tumour of Oddi introduced by Marchal and Hureau[1]. The sphincter of Oddi exactly delineates the junction between the bile duct, pancreatic duct and duodenum. We wanted to avoid using the anatomic term ampulla of Vater, since this structure rarely appears as an ampulla. This then excludes tumours in the head of pancreas, common bile duct above ths phincter of Oddi and tumours of the duodenal wall adjacent to the papilla. These tumours seem to behave differently from other pancreatic tumours, as they carry a different prognosis and need special attention. We have therefore reviewed retrospectively 56 patients with tumours of Oddi with special reference to diagnosis, histopathologic examination and surgical therapy.

KEY WORDS: Tumours of Oddi, Whipples' procedures, ampullectomy.

### MATERIAL AND METHOD

Fifty-six patients were operated upon for ampullary tumours at the Centre de Chirurgie Digestive de l'Hopital Saint Antoine during the 15 years ranging from

Address correspondence to: B. Nordlinger, M.D., Centre de Chirurgie Digestive de l'Hopital Saint-Antoine, 184 rue du Faubourg Saint-Antoine, F-75012 Paris, France.

*Present address: Department of Surgery, Lund University, S-221 85 LUND, Sweden.

October 1970 to October 1985. The clinical histories, operative findings and histologic specimens were reviewed. All patients were followed-up, except five, where information was retrieved from charts, referring physicians or the civil registry.

### RESULTS

### *Clinical Signs*

Obstructive jaundice was the most frequent presenting symptom (84%). It was progressive and evident in 25 patients, intermittent in seven patients or associated with cholangitis in 16. Weight loss exceeding 10% of the body weight was observed in 20 patients (36%). Twenty patients had abdominal pain (36%). The pain was remitting, of low intensity and had persisted for several months. Twelve patients (21%) had hypochromic anemia, and five out of these patients had signs of gastrointestinal bleeding. Seventeen patients exhibited hepatomegaly (30%), a distended gallbladder was noted in 12 (21%) and finally a palpable mass in the epigastrium was noted in three patients.

### *Diagnostic Work-up*

Bilirubin levels ranged from 68 to 340 umol/l in patients with obstructive jaundice, except in nine where bilirubin was normal on admission.

The diagnostic approach changed during the 15 years of the study. Among 19 patients having a barium meal, only five exhibited a diagnostic impression on the second part of the duodenum. At duodenoscopy the tumour of Oddi was evident in 12 patients and 7/12 biopsies were positive. Endoscopic retrograde cholangio pancreatography (ERCP) revealed the tumours in 7/7 patients. Twenty-three patients had abdominal ultrasound but only 12 were diagnostic in that they showed dilated bile ducts with an echogenic mass protruding into the duodenal lumen. In five patients a probable diagnosis of pancreatic cancer was made. In two patients the likely diagnosis of common bile duct stone was made and in the remaining four patients dilated bile ducts were observed without any obvious etiology. A PTC (percutaneous transhepatic cholangiography) showed in one patient an obstruction at the level of the sphincter. Arteriography (four patients), intravenous cholangiography (one patient), liver scintigraphy (two patients) or laparoscopy (seven patients) were of no diagnostic value.

### *Histopathologic Examination*

Seven of the twelve biopsies taken at endoscopy were diagnostic. All other diagnoses were made at laparotomy. The tumour mass ranged from 0.5 - 5 cm (mean 2.3 cm). Two thirds of the tumours were exophytic (the largest of 5 cm was growing intraduodenally) and one third were infiltrating.

Fifty-one (91%) were adenocarcinomas. Forty five were well differentiated, two thirds had a papillary or villous growth pattern and they were especially extending into the duodenum. One third consisted of tubulo-alveolar or tubulo-excretory cancers with infiltration in the duct and the duodenal wall. The latter group of tumours were macroscopically infiltrating and ulcerating. Six tumours were anaplastic or undifferentiated. Lymph node metastases were present in 18 cases. According to the classification of Martin[2] (Table 1) five tumours were stage I, 10 stage II, 18 stage III and 18 stage IV>

Five tumours were benign: three adenomas were operated upon with a pancreaticoduodenal resection, resection of the ampulla and local excision with sphincterotomy respectively; two hyperplastic polyps were removed by local excision and sphincterotomy.

**Table 1** Histologic classification of tumours of Oddi according to Martin

| | |
|---|---|
| Stage I | Local tumour, intra-ampullary or duodenal with a papillary or villous growth and without infiltration of underlying structures. |
| Stage II | Infiltration of the sphincter of Oddi or bile duct or the submucosa of the duodenum without penetration of the muscular layer of duodenum. |
| Stage III | Infiltration at or beyond the muscular layer of the duodenum. |
| Stage IV | Regional or distant lymph node metastases or extension periduodénally or into pancreas. |

### *Surgical Therapy*

All patients were operated upon (Table 2). Nine patients underwent a local resection: six ampullectomies and three local excisions with sphincterotomy. The six ampullectomies were performed in two cases for a benign tumour; one for a malignant tumour, three for malignant tumours subsequently followed by pancreaticoduodenal resection (two had local recurrencies 14 and 24 months later and one patient had incomplete local tumour excision). These last three patients appear in the group of pancreatico-duodenal resections. One ampullectomy for a malignant tumour was followed by a choledocho-duodenostomy because of difficulties in reimplantation of the common bile duct in the duodenum.

The three local excisions of the benign tumours were performed without complete ablation of the sphincter and followed by a simple sphincterotomy.

**Table 2** Mortality and morbidity

| | *Number* | *Mortality* | *Morbidity* |
|---|---|---|---|
| Local excision* | 9 | 0 | 0 |
| Duedeno-pancreatic resection: | | | |
| Total | 41 | 3 (7.3%) | 12 (29%) |
| Primary | 28 | 3 (7.3%) | 9 (32%) |
| Secondary | 13 | 0 | 3 (23%) |
| By pass | 10 | 1 (10%) | 0 |
| Total* | 56 | 4 (2.2%) | 12 (21.5%) |

* Three cases with local excision underwent a subsequent duodeno-pancreatic resection and one a bypass procedure.

Fourty-one patients had a pancreatico-duodenal resection. Twenty-eight were performed as a primary procedure, 10 after pre-operative biliary decompression and three following previous ampullectomy. One of these tumours was benign and 40 were malignant.

Ten patients had only a bilio-enteric bypass for malignant tumours. The morbidity and mortality are given in Table 2. There were four hospital deaths: one cardiac arrest in a 89 year-old lady after a biliary diversion and three deaths after pancreatico-duodenal resections; two with a leaking pancreatico-jejunostomy and one with a bleeding anastomotic ulcer.

There were no complications in patients undergoing biliary diversion alone or local resection. In the group of pancreatico-duodenal resections there were complications in 12 patients (29%): six pancreatic fistulas with an output ranging from 0.5 – 1.5 1/24 h and lasting 20-45 days; one peritonitis after breakdown of the pancreatico-jejunal anastomosis; three gastrointestinal hemorrhages from diffuse gastritis; one prolonged gastric retention and one wound abscess. There was one late complication with an anastomotic ulcer after six months.

Thirty-one of the fifty-six patients have died during the observation period of 15 years. Besides the four reoperations mentioned above, seven patients had liver metastases, seven local tumour extensions, three peritoneal carcinomatosis, one cerebral metastases and one had pulmonary metastases. In eight patients the cause of death is unknown. Forty-one percent of the patients were alive at five years after a pancreatico-duodenal resection for malignant tumours and 10% of the patients after biliary diversion alone (Figure 1). Life table analysis according to tumour stage shows that 75% of the patients were alive at five-years in stage I, 57% in stage II, 33.6% in stage III and 8% in stage IV (Figure 2).

The three patients who underwent ampullectomy alone, one for a benign and two for malignant tumours are alive and well at one, two and three years respectively. Among the five patients with benign tumours, one died after a subsequent cardiac operation, one is lost to follow-up. The others are alive and well at one, three and six years respectively.

### DISCUSSION

Jaundice was the most common presenting symptom of the patients with tumours of the Oddi. Most often it was clinically evident and of the type seen in pancreatic cancer, sometimes as seen in cholangitis. A variable and recurrent jaundice was seen in only 12% of the patients. In 36 percent of the patients it had been preceded by intermittent abdominal pain, as has been reported in other series as well[3,4,5]. The combination of jaundice and gastrointestinal bleeding was rapidly diagnostic, but it was not frequent in this study, where only 10% had melaena and 21% anemia[6]. In the literature the frequency ranges from 25 to 60%[1]. Some of the patients had had symptoms for more than six months, which could indicate a slow progression of the tumour[7] or the development from an initially benign tumour[8,9].

The most widely used means of confirming the diagnosis today are ultrasound, ERCP and PTC. Ultrasound is the first examination in cases of obstructive jaundice. It usually shows dilated bile ducts and the site of obstruction. To prove the presence of a tumour of Oddi is more difficult and depends on the experience of the examiner, the morphology of the tumour and its size, since tumours of less than

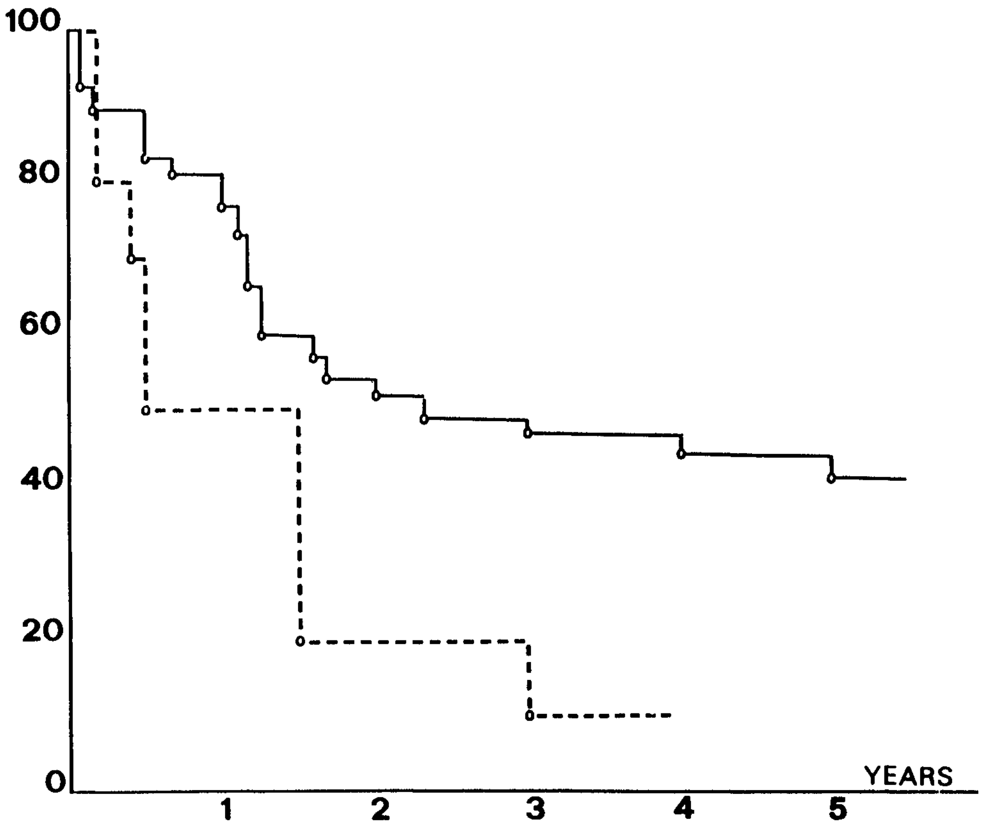

**Figure 1** Surgical treatment of tumours of Oddi. Life table analysis (Kaplan-Meier) of survival after treatment of 50 tumours.
(————————) n = 40 duodeno-pancreatic resection
(. . . . . ) n = 10 by pass
Patients undergoing local resection alone are not included in the figure.

one cm diameter are rarely seen. It may be possible to detect smaller tumours by the use of endoscopic ultrasound. In the present series ultrasound was diagnostic in more than half of the cases preoperatively.

Sideviewing duodenoscopy is the preferred method of examination[10]. It allows direct vision of tumours of the Oddi and their intraduodenal growth, which was found in 2/3 of the cases in our series and it also makes biopsy possible. In this series 7/12 biopsies confirmed the diagnosis and 42% were false negatives. Walsh reported 25% false negatives in his series[11]. Nakao[12] reviewed 26 series of ampullary tumours of a total of 460 cases and found that in only 3.7% was the diagnosis reached preoperatively. Even with progress in endoscopic techniques one can only count on the positive biopsies and only be certain that a tumour is benign (<10 percent of cases) when examining the excised tumour. ERCP may increase the

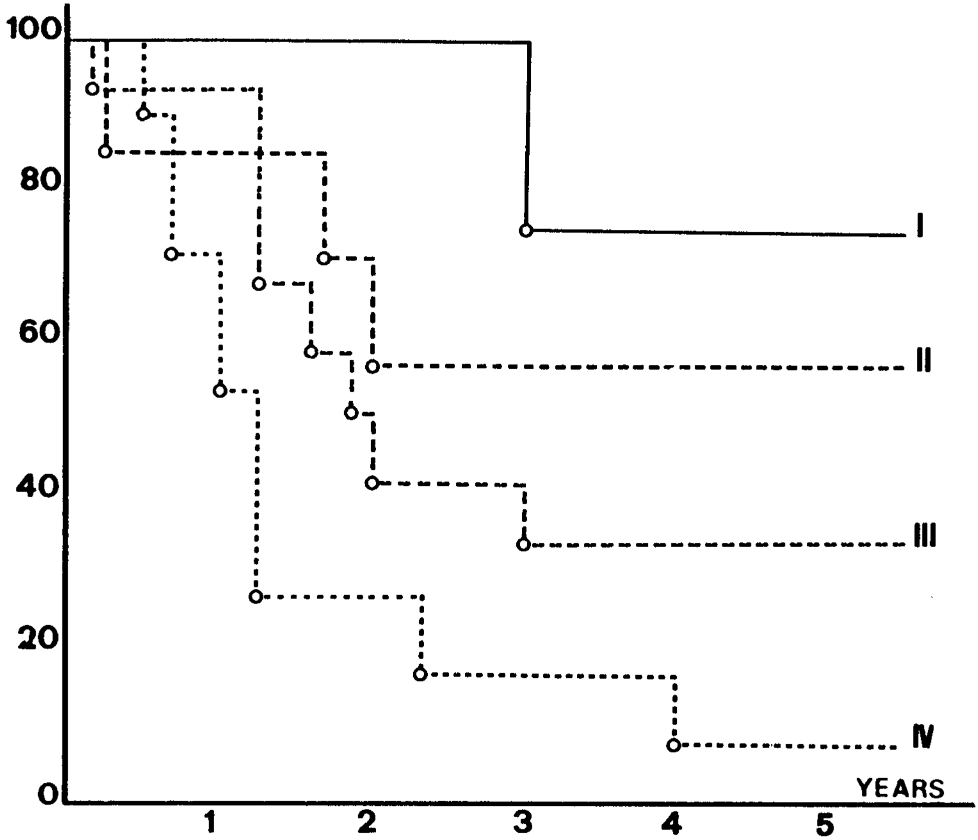

**Figure 2** Survival curves (Kaplan-Meier) after duodeno-pancreatic resection (n = 40) according to tumour stage. The three operative deaths are excluded. Stage I :n = 5. Stage II :n = 8. Stage III :n = 16. Stage IV :n = 11.

diagnostic accuracy to 85-90%[1,13]. Seyrig *et al.* performed an endoscopic sphincterotomy to aid diagnosis and to obtain preoperative biliary drainage[14]. Percutaneous transhepatic cholangiography is preferred by some authors and this allows for easy determination of the proximal extent of the tumour[4]. It also allows preoperative biliary drainage. It carries a significant morbidity though[15] and we think that it is not necessary in most instances. The suspicion of a tumour of Oddi in most cases, leads to operative intervention. At operation cholangiography, choledochoscopy or duodenotomy will ascertain the diagnosis. Frozen section biopsies are difficult to interpret and are accompanied by a failure rate of 10 to 35%[10,16,17,18]. In any case, the presence of a tumour of Oddi should lead to an excision, since the majority are malignant (91% in this material). Moreover, the rare benign lesions (adenoma and hyperplasia), are considered to be premalignant[19]. In a recent investigation adenomatous structures were found in the center of malignant tumours of Oddi in 91.4%[20].

Surgical resection alone allows for long term survival and duodeno-pancreatic resection is the preferred operation, undertaken in 73% of the patients in the present series. The preoperative biliary drainage is a very controversial issue[15,21,22] and seems often of little value. The overall operative mortality in this series was 7.1 percent and 7.3 percent after duodeno-pancreatic resection. Higher mortality figures of around 20% are found in older publications[23,24], but more recent reports have shown an improvement[10,25,26]. We have also observed a continuous improvement and only had one death in the last 50 duodeno-pancreatic resections.

The main cause of morbidity and mortality is dehiscence in the pancreatico-jejunal anastomosis[17,22,23,25]. The improved technique reported by our group[27] and which leaves a jejunal segment of 60 cm between the pancreatico-jejunal and biliary anastomosis, avoids the risk of bile reflux into the pancreatico-jejunal anastomosis and transforms a potential pancreatico-jejunal dehiscence into a pure pancreatic fistula, which is of lesser gravity.

The overall five years survival after duodeno-pancreatic resection for a malignant tumour of the ampulla of Vater has been 41%, depending on the degree of local invasion. The classification of Martin[2] (Table 2) allows for a better estimation of prognosis after resection. It includes the localisation, histologic type, size and lymph node involvement. According to this, tumors of stage I, which are localized and non-invasive, have a five year survival of 75-80%. Corresponding figures for stage II (not growing beyond the submucosa of the duodenum) and stage III (penetrating tumours) are 40-20%. The figures are better in this series, but the number of patients in the different groups is fairly small. In stage IV tumours with evident lymph node metastases, the five year survival is less than 10%[9].

After bilio-enteric anastomosis alone, less than 50% of the patients are alive after six months and only one lives for three years. These poor results have been confirmed by other studies as well[10].

Halsted[28], described in 1899 duodenopapillectomy or ampullectomy. This operation consists of a local resection of the tumour and the sphincter of Oddi with surrounding duodenal wall via a duodenotomy. It necessitates the reinsertion of the common bile duct and pancreatic duct into the duodenum, which can be a delicate task. This operation may be sufficient in cases of benign tumours, where the histologic examination of the entire tumour embedded in paraffin blocks has excluded the presence of foci with cancer growth. It is however insufficient to cure malignant tumours of Oddi, which constitute the majority of the cases. Of the five patients with a malignant tumour of Oddi who had a local resection in this series, three had to be reoperated with a duodenopancreatic resection subsequently. This operation may be applied in patients unfit for a duodeno-pancreatic resection and low mortality rates have been reported by several groups[29,30,31]. It allows an effective drainage of the bile duct and pancreatic duct into the duodenum and also avoids the continuing bleeding from the tumour. This procedure thus has advantages compared to bilio-enteric diversion alone or endoscopic treatment. This was exemplified in one 79 year old lady in this material where biliary drainage and hemostasis was achieved. Two years later she had an intraluminal recurrence, which could not be locally excised, but then treated by a duodeno-pancreatic resection, which was well tolerated.

The duodeno-pancreatic resection is thus the preferred operation for the majority of these tumours and the ampullary resection should be used only exceptionally. There are no reports of adjuvant treatments such as radiotherapy or chemotherapy being of any benefit for tumours of Oddi.

### *References*

1. Marchal, G. and Hureau, J. (1978) Les tumeurs oddiennes (ampullomes vatériens), Monographie de l'Association Fran<cc>aise de Chirurgie. Paris: Masson pp. 49–50, 35–51
2. Martin, E.D. (1977) The sphincter of Oddi. Basel: S.Karger
3. Catell, R.B., Warren, K.W. and Au, F.T.C. (1959) Peri-ampullary carcinomas; diagnosis and surgical management. *Surg. Clin. North Am.*, **39**, 781–798
4. Diard, F., Drouillard, J. Gouffand, J.M., Rabin, A. and Delorme, G. (1975) Radiologie des tumeurs de la région vatérienne. A propos de 18 cas. *J. Radiol. Electrol.*, **56**, 307–315
5. Monge, J. and Dockerty, M. (1964) Clinicopathologic observations in radical pancreaticoduodenal resection for peri-ampullary carcinoma. *Surg. Gynecol. Obstet.*, **118**, 275–283
6. Sandblom, P. and Mirkovitch, V. (1979) Minor hemobilia. Clinical significance and pathophysiological background. *Ann. Surg.*, **190**, 254–264
7. Bergeret, A., Caroli, J. and Lacaux, J. (1948) Ampullome vatérien. Diagnostic radiomanométrique. Duodénopancréatectomie. Guérison. *Arch. Mal. App. Dig.*, **3**, 244–249
8. Leonard, P. (1972) L'ampullome vatérien. Aspects evolutifs et problémes diagnostiques (é propos de 6 observations d'affections au long cours). *Rev. Fr. Gastroenterol.*, **81**, 5–36
9. Yamaguchi, K. and Enjoji, M. (1987) Carcinoma of the Ampulla of Vater. A Clinicopathologic study and pathologie staging of 109 cases of carcinoma and 5 cases of adenoma. *Cancer*, **59**, 506–515
10. Neoptolemos, J.P., Talbot, I.C., Carr-Locke, D.L., Shaw, D.E., Cockleburgh, R., Hall, A.W. and Fossard, D.P. (1987) Treatment and outcome in 52 consecutive cases of ampullary carcinoma. *Br. J. Surg.*, **74**, 957–961
11. Walsh, D. Eckhauser, F., Cronenwett, J. Turcotte, J. and Lindenaver, S. (1982) Adenocarcinoma of the ampulla of Vater. Diagnosis and treatment. *Ann. Surg.*, **195**, 152–157
12. Nako, N. Siegel, J., Stenger, R. and Gelb, A. (1982) Tumors of the ampulla of Vater: Early diagnosis by intra-ampullary biopsy during endoscopic cannulation. *Gastroenterology*, **83**, 459–464
13. Hall, T., Blackstone, M., Cooper, M., Hughes, R. and Moossa, A. (1978) Prospective evaluation of endoscopic retrograde cholangiopancreatography in the diagnosis of per-ampullary cancers. *Ann. Surg.*, **187**, 313–317
14. Seyrig, J.A., Liguory, C., Meduri, B., Ink, O. and Buffet, C. (1983) Endoscopie dans les tumeurs de la région oddienne. Possibilités diagnostiques et thérapeutiques. *Gastroenterol. Clin. Biol.*, **7**, 854–867
15. MacPherson, G.A.D., Benjamin, I.S., Habib N.A., Bowley, N.B. and Blumgart, L.H. (1982) Percutaneous transhepatic drainage in obstructive jaundice: advantage and problems. *Br. J. Surg.*, **69**, 261–264
16. Kellum, J., Clark, J. and Miller, J. (1983) Pancreaticoduodenectomy for resectable malignant periampullary tumours. *Surg. Gynecol. Obstet.*, **157**, 363–366
17. Warren, K.W., Choe, D.S., Plaza, J. and Relihan, M. (1975) Results of radical resection for periampullary cancer. *Ann. Surg.*, **181**, 534–540
18. Warren, K.W. and Hoffman, G. (1976) Changing patterns in surgery of the pancreas. *Surg. Clin. North Am.*, **56**, 615–629
19. Bergdahl, L. and Andersson, A. (1980) Benign tumours of the papilla of Vater. *Ann. Surg.*, **46**, 563–566
20. Baczako, K., Buchler, M., Berger, H.G., Kirkpatrick, J. and Hafferkamp, O. (1985) Morphogenesis and possible precursor lesions of invasive carcinoma. *Hum. Pathol.*, **16**, 305–310
21. Fortner, J., Kim, O., Cubilla, A., Turnbull, A., Pahnke, L. and Sihls, M. (1977) Regional pancreatectomy. En bloc pancreatic, portal vein and lymph node resection. *Ann. Surg.*, **186**, 42–50
22. Gilsdorf, R. and Spanos, P. (1977) Factors influencing morbidity and mortality in pancreaticoduodenectomy. *Ann. Surg.*, **177**, 332–337
23. Nakase, A., Matsumoto, Y., Uchida, K. and Honjo, I. (1977) Surtical treatment of cancer of the pancreas and the peri-ampullary region. Cumulative results in 57 institutions in Japan. *Ann. Surg.*, **185**, 52–57
24. Wise, L., Pizzimbond, C. and Dehner, L. (1976) Peri-ampullary cancer: a clinocopathologic study of sixty-two patients. *Am. J. Surg.*, **131**, 141–148
25. Barton, R. and Copeland, E. (1983) Carcinoma of the ampulla of Vater. *Surg. Gynecol. Obstet.*, **156**, 207–310
26. Jones, B., Langer,B., Taylor, B. and Girotti, M. (1985) Peri-ampullary tumours; which one should be resected? *Am. J. Surg.*, **149**, 46–52

27. Parc, R. and Herbiére, P. (1983) Protection de l'anastomose pancréatico-jéjunale aprés duodéno-pancréatectomie céphalique pour tumeur. *Nouv. Presse Med.*, **12**, 99–101
28. Halsted, W. (1889) Contributions to the surgery of the bile passages especially the common bile duct. *Boston. Med. Surg. J.*, **141**, 646–654
29. Newman, R.J. and Pittam, M.R. (1982) Local excision in the treatment of carcinoma of the ampulla of Vater. *J. R. Coll. Surg. Edinb.*, **27**, 154–157
30. Goldberg, M., Zamir, D., Hadary, A. and Nissan, S. (1987) Wide local excision as an alternative treatment for peri-ampullary carcinoma. *Am. J. Gastroent.*, **82**, 1169–1171
31. Isaksson, G., Ihse, L. and Andrén-Sandberg, A. (1982) Local excision for ampullary carcinoma. An alternative treatment for patients unfit for pancreatectomy. *Acta Chir. Scand.*, **148**, 163–165

*(Accepted by S.Bengmark 27 July 1991)*

### INVITED COMMENTARY

The authors have reported on their experience of 56 patients with tumours of Oddi and are to be congratulated not only on their excellent surgical results but also in their analytical approach.

The authors have commented on the increasing role of endoscopic retrograde cholangiopancreatography (ERCP) in the diagnosis of these tumours. It is apparent that improvements in abdominal ultrasonography have also taken place as in 12 of 23 cases (52%) a precise diagnosis was made using this modality alone.

Biopsies were positive in only seven of 12 cases (58%) in whom this was undertaken at ERCP. This diagnostic problem has previously been reported by Leese *et al.*[1]. Tumours of Oddi may be confused macroscopically with inflammatory pseudotumours either at ERCP or during surgery. The authors are correct in indicating that frozen section is often not reliable and that paraffin section histology is required. The diagnostic yield can be increased by undertaking a biopsy of the centre of the tumour mass *after* endoscopic sphincterotomy, if need be repeating the procedure[1]. One would agree with the authors that if there is any doubt a formal resection should be undertaken.

Although there is a general consensus of opinion that adenomas of this area are premalignant, there is little substantial evidence that this applies to "hyperplastic" lesions. Indeed, such lesions may represent a variant of the inflammatory pseudotumour which appears to be quite benign and which often regresses[1].

The authors' lack of enthusiasm for local resection of these tumours is shared by others. Although the operative mortality rate was low in their hands and is similarly low in a few series, others report a very high mortality. Although some have recommended this as the primary treatment[2], this has little validity as these tumours have not been staged and therefore survival cannot be properly judged in comparison to the Whipple operation.

The authors are to be congratulated for analysing their data according to Martin[3]. The official TNM system does not appear to sufficiently categorise outcome for patients who have undergone resection. Unfortunately, the system of Martin has received little recognition but a number of groups have come up with a very similar system to that of Martin[4-7]. Clearly it should be one of the tasks of the hepato-biliary community to agree on a standardized system of staging.

The main development of endoluminal (endoscopic) US is perhaps not so much in diagnosis but in the accurate pre-operative staging of tumours of Oddi[8]. In this way it may be possible to choose the best treatment for each individual patient.

The operative mortality mirrors the general trend in specialised units in achieving a commendably low rate with figures improving even more recently. It should also be borne in mind that endoscopic sphincterotomy (with or without stenting) can produce excellent palliation in high risk cases with some five year survivors even in biopsy proven cases[9,10].

Although no forms of adjuvant therapy have yet to be shown to have any value, it is apparent that patients with Martin's Stages III and IV represent a group at whom future trials should be aimed. Encouraging results have been obtained with radiotherapy and 5-fluorouracil in pancreatic cancer[11] and such a regimen may be suitable in cases of tumours of Oddi. Multicentre trials will be required in order to resolve this.

Finally, pylorus-preserving pancreatico-duodenectomy is emerging as an important development in the treatment of tumours of Oddi. Although the results are still relatively early at the present, this procedure seems to offer improved well-being to patients without compromising long-term survival[12].

### *References*

1. Leese, T., Neoptolemos, J.P., West, K.P., Talbot, I.C. and Carr-Locke, D.L. (1986) Tumours and pseudotumours of the region of the ampulla of Vater: an endoscopic, clinical and pathological study. *Gut*, **27**, 1186–1192
2. Knox, R.A. and Kingston, A.D. (1986) Carcinoma of the ampulla of Vater. *Br. J. Surg.*, **73**, 72–73
3. Martin, E.D. (1977) The sphincter of Oddi. Basel: S. Karger
4. Baczako, K., Buchler, M., Beger, H.G., Kirkpatrick, C.J. and Haferkams, A.O. (1985) Morphogenesis and possible precursor lesions of invasive carcinoma of the papilla of Vater: Epithelial dysphasia and adenoma. *Hum. Pathol.*, **16**, 305–310
5. Dinges, H.P. and Sellner, F. (1981) Zum "staging" und "Grading" von karzinomen on der papilla Vater. *Wien Klin. Wschr.*, **93**, 638–643
6. Neoptolemos, J.P., Talbot, I.C., Shaw, D.E. and Carr-Locke, D.L. (1988) Long-term survival following resection of ampullary carcinomas is independently associated with tumour grade and a new staging classification which assesses local invasiveness. *Cancer*, **61**, 1403–1407
7. Yamaguchi, K. and Enjoji, M. (1987) Carcinoma of the ampulla of Vater. A clinicopathologic study and pathologic staging of 109 cases of carcinoma and 5 cases of adenoma. *Cancer*, **59**, 506–515
8. Tio, T.L. and Tytgat, G.N.J. (1986) Endoscopic ultrasonography in staging local resectability of pancreatic and periampullary malignancy. *Scan. J. Gastroenterol.*, **21**, 135–142
9. Neoptolemos, J.P., Talbot, I.C., Carr-Locke, D.L., Shaw, D.E., Cockleburgh, R., Hall, A.W. and Fossard, D.P. (1987) Treatment and outcome of 52 consecutive case of ampullary carcinoma. *Br. J. Surg.*, **74**, 916–917
10. Bickerstaff, K.I., Berry, A.R., Chapman, R.W. and Britton, B.J. (1990) Endoscopic sphincterotomy for the palliation of ampullary carcinoma. *Br. J. Surg.*, **77**, 160–162
11. GITSG (1987) Further evidence of effective adjuvant combined radiation and chemotherapy following curative resection of pancreatic cancer. *Cancer*, **59**, 2006–2010
12. Grace, P.A., Pitt, H.A. and Longmire, W.P. (1990) Pylorus preserving pancreatoduodenectomy: an overview. *Br. J. Surg.*, **77** (in press)

J.P.Neoptolemos

Dept. of Surgery

University of Birmingham

### INVITED COMMENTARY

This retrospective review of 56 patients operated upon for tumors of Oddi over a 15 year period presents some interesting and perplexing problems. The analyses regarding clinical and laboratory features, diagnostic procedures employed and pathologic analysis of the material was carefully conceived and taken together, yield anticipated and acceptable conclusions.

I am perplexed by the definitation and limitation of the anatomic term Oddi, although it has been defined by Marchal, Hureau and others.

If the area covered by the sphincters described by Oddi and refined by many surgeons and anatomists, especially by Boyden[1], the terminal bile duct and the terminal duct of Wirsung are a part of the Oddi apparatus. Thus the precise origin of these tumors must be variable, and at times, difficult to determine.

Despite this confusion on my part this manuscript contains important data and valid conclusions. The operative mortality is within the currently acceptable range.

The incidence of pancreatojejunal fistula was high but the authors indicated that they had altered their technique of this anastomosis with favorable results. A precise description of their current method would have been revealing.

Their final conclusion that pancreatoduodenectomy is the preferred operation in the treatment of these tumors is correct.

### *References*

1. Bowden, E.A. (1957) The sphincter of Oddi in man and in certain representative mammals. *Surgery*, **1**, 25–37

K.W.Warren

North East Baptist Hospital

Boston

Mass., USA

